# Factors associated with blood donation among college and university students in Wuhan, China: structural equation model

**DOI:** 10.1186/s12889-024-19384-y

**Published:** 2024-07-10

**Authors:** Mengdi Ma, Ru Yang, Jun Gu, Songqing Ke, Xiaoan Du, Jie Zheng

**Affiliations:** Wuhan Blood Center, Wuhan, China

**Keywords:** Blood donation, Student, Health status, Structural equation modeling, China

## Abstract

**Background:**

College and university students were an important population group of blood donors, especially in the current situation of tight blood supply. This study aimed to investigate the current status and determinants of blood donation among this population group in Wuhan using a structural equation model.

**Methods:**

We conducted a cross-sectional study involving 12 colleges and universities in Wuhan, China, including 5168 students. Sociodemographic characteristics, health status, knowledge about blood donation, and attitude toward blood donation were treated as latent variables, with blood donation as the observed variable. Confirmatory factor analysis was conducted using the Mplus 8.0 statistical software application, followed by the establishment of a structural equation model to assess the relationships that exist between these variables.

**Results:**

The overall blood donation rate among college and university students was 24.71%. The established model indicated that sociodemographic characteristics, health status, knowledge about blood donation, and attitude toward blood donation showed significant positive effects (0.135, 0.056, 0.321, and 0.389, respectively) on blood donation, among them, the direct effects were 0.076, -0.110, 0.143, and 0.389, respectively (*P* < 0.01). Additionally, sociodemographic characteristics, health status, and knowledge about blood donation had indirectly affected blood donation through the mediating effect of attitude towards blood donation. Their effects on attitude towards blood donation were 0.099, 0.243, and 0.468, respectively. (*P* < 0.01). The model could explain explained 22.22% of the variance in blood donation behavior among college and university students in Wuhan.

**Conclusion:**

Blood donation among college and university students in Wuhan was associated with sociodemographic characteristics, health status, knowledge about blood donation, and attitude towards blood donation, with attitude being the primary influencing factor. Tailored recruitment strategies for blood donation among students should prioritize initiatives aimed at enhancing knowledge about blood donation and fostering positive attitudes toward it.

**Supplementary Information:**

The online version contains supplementary material available at 10.1186/s12889-024-19384-y.

## Introduction

Blood donation is indispensable in clinical treatment, with all clinical blood in China-sourced from voluntary unpaid donors. However, China’s current blood donation rate stands at 12‰, significantly lower than rates in high-income countries (31.5‰) and upper-middle-income countries (16.4‰) according to the World Health Organization [1]. A survey on blood collection and supply across China in 2017 revealed a national demand surpassing supply, exacerbating the supply-demand disparity [2]. With the aging population and rising healthcare needs, enhancing blood donation rates and supply becomes imperative.

College and university students, often in good physical health and receptive to blood donation, represent a high-quality donor group [3, 4]. Lin et al.’s study identified a direct correlation between college enrollment and blood donation rates among individuals aged 18–34, emphasizing the pivotal role of education, particularly university education, in donation propensity [5]. Despite their potential, significant variations in blood donation [3, 6] rates among college or university students exist across different Chinese cities (e.g., Jinan, 50.22%; Fuzhou, 38.67%; Shaoxing, 7.33%) [7–9], mirroring global disparities [3, 4, 10–12], indicating a universal blood donation strategy may yield limited effectiveness. Moreover, the COVID-19 pandemic has adversely affected college students’ blood donation behaviors, potentially due to compromised physical or mental health conditions [13, 14]. These findings underscore the need for tailored interventions targeting college and university students, emphasizing the urgency of addressing blood donation disparities in this key demographic.

The study of factors associated with blood donation behavior among college and university students can provide critical insights for developing effective recruitment strategies. Researches have identified connections between blood donation among university students and various factors such as gender, major, religion, descriptive norms, intention, knowledge, and social morality [4, 15, 16]. However, the existing findings remain certain limitations. Firstly, blood donation necessitates certain physical health criteria and is linked to promoting mental well-being [17, 18]. Nevertheless, scant attention has been paid to the impact of health status on blood donation in existing research, particularly in light of the COVID-19 pandemic where health status may exert a more pronounced influence [19, 20]. Above and beyond, most previous studies relied on regression analysis, unable to fully elucidate the structured relationships among multiple variables.

Thus, this study aims to enhance the analysis of blood donation among college and university students by integrating self-assessed health status—encompassing physical, mental, and social dimensions—into the investigation of potential influencing factors. Utilizing Structural Equation Modeling (SEM), a multivariate analysis technique that simultaneously estimates the structural links between independent and dependent variables [21, 22], we aim to discern direct, indirect, and total effects of multiple variables on blood donation behavior among college and university students in Wuhan.

As a major medical center in central China, Wuhan faces a significant challenge in balancing clinical blood demand with supply. Given that Wuhan has one of the largest student populations in the country, with over 1.3 million college and university students, developing targeted strategies to recruit these students as blood donors is crucial to alleviate the regional blood supply-demand tension. This study could offer a scientific basis for such strategies. Additionally, the findings could serve as a reference for the recruitment of blood donors from college and university students in other regions.

## Methods

### Study design and population

A cross-sectional study was conducted in Wuhan, China, and it received approval from the Ethics Committee of Wuhan Blood Center. A two-stage sampling method was performed from November 22th to December 12th 2021. Initially, 8 universities (comprising 4 ordinary universities and 4 independent universities) and 4 colleges were selected from the total of 83 universities and colleges in Wuhan, based on educational level and school-running entities. Trained investigators, sourced from the volunteer organizations within the selected colleges and universities, conducted the online survey on their respective campuses. Inclusion criteria for participants: (1) College and university students in Wuhan; (2) Voluntary participation. Exclusion criteria: (1) Under 18 years old; (2) Having a reading disability. Each participant was required to sign an electronic informed consent form before responding to the survey questions. A total of 5,174 qualified questionnaires were gathered, and after excluding those with rapid submissions or missing values for main outcome variables, 5,168 participants were retained in the final sample for onward analysis. The sample size was deemed sufficient.

### Data collection tools and method

A self-administered questionnaire was developed in Chinese, and underwent two rounds of revision after reviewed by two experienced blood bank staff members and one public health expert. It mainly comprised five sections: sociodemographic characteristics, health status, knowledge about blood donation, attitude toward blood donation, and blood donation behavior. The translated version is shown in Supplementary Material 1. Validity analysis demonstrated a KMO value of 0.725 and a Bartlett’s test result with a *P* value of < 0.001. The questionnaire was distributed to students through a QR code on WeChat APP. To ensure the completion and quality of the questionnaire, stringent format guidelines and skip logic rules were implemented.

### Study variables

In line with the study’s objectives and our literature review, we aim to investigate the structural relationships between blood donation behavior and four latent variables: demographic characteristics, health status, knowledge about blood donation, attitude towards blood donation. The demographic characteristics factor encompasses gender, age, level of education, political stance, and monthly living expenses. Health status was assessed using the Self-rated Health Measurement scale—the Revised Version 1.0 (SRHMS-V1.0)—comprising three dimensions: physical health status, mental health status, and social function [23]. The Cronbach’s alpha of this scale among Chinese college students was 0.940, and it was 0.886 in this study [24]. Knowledge about blood donation, attitude towards blood donation and blood donation behavior were evaluated using self-designed items. Supplementary Table 1 outlines the hypothetical latent variables and their corresponding observational variables.

### Statistics

Initially, descriptive statistics were performed using SPSS version 22.0 statistical software application to characterize the participants’ basic traits and blood donation behaviors. Subsequently, latent variable analysis, incorporating confirmatory factor analysis (CFA) and SEM, was conducted using Mplus version 7.4 statistical software application. Our use of CFA is premised on the empirical need to validate the effective measurement of latent variables by the selected observational variables. Building upon the measurement model established by CFA, SEM was employed to examine the associations between latent variables and blood donation behavior. All observational variables were categorized as categorical variables during model estimation. Likewise, the parameter estimation for CFA and SEM utilized the weighted least squares with mean and variance (WLSMV)-adjusted method, with effects quantified through standardization. Four goodness-of-fit indices—chi-square (χ2) and degrees of freedom (*df*), comparative fit index (CFI), Tucker–Lewis’s index (TLI), and root mean square error of approximation (RMSEA)—were employed to assess the fit of the hypothesized model to the data. Notably, a large sample size can yield statistically significant results for the chi-square test, hence, caution was exercised [25]. Besides, a well-fitted model was indicated by both CFI and TLI values exceeding 0.90, and RMSEA below 0.08 [25, 26], whereas, statistical significance was set at a two-sided *P* < 0.05 for all analyses.

## Results

### Participants and blood donation

Table 1 illustrates the demographic characteristics and blood donation prevalence among the participants. Out of the 5,168 participants, 48.45% were male (*n* = 2,504). The average age was 18.93 ± 1.46 years old, with about 78.27% falling within the 18–19 age bracket. 38.74% of the participants hailed from colleges, while 77.19% were affiliated with either the Chinese Communist Youth League or the Communist Party of China. Also, urban hukou status was held by 37.35% of the participants, and 61.01% were the only children in their families. Moreover, 84.38% of participants reported monthly living expenses exceeding 1,000 RMB. Regarding blood donation prevalence, Table 1 indicates a total rate of 24.71%. Equally, chi-square test results revealed significant variations in blood donation prevalence among participants based on gender, age, political affiliation, hukou type, in only-child family or not and monthly living expenses.

**Table 1:** Characteristics of participants and blood donation

Variable	*N* (%)	Blood donation (*N* / prevalence %)	χ^2^	*P*
**Gender**			30.992	< 0.001
Male	2504 (48.45)	705 (28.15)		
Female	2664 (51.55)	572 (21.47)		
**Age (years old)**			213.063	< 0.001
18	2357 (45.61)	386 (16.38)		
19	1688 (32.66)	458 (27.13)		
20	685 (13.25)	250 (36.50)		
≥ 21	438 (8.48)	113 (41.78)		
**Education being received**			2.892	0.089
College	2002 (38.74)	469 (23.43)		
University	3166 (61.26)	808 (25.52)		
**Politic countenance**			16.174	< 0.001
Members of the CCYL or CPC^†^	3989 (77.19)	1038 (26.02)		
Other parties or masses	1179 (22.81)	239 (20.27)		
**Type of Hukou**			7.076	0.008
Urban	1930 (37.35)	437 (22.64)		
Rural	3238 (62.65)	840 (25.94)		
**Only-child family**			6.281	0.012
Yes	3153 (61.01)	817 (25.91)		
No	2015 (38.99)	460 (22.83)		
**Monthly living expenses**			8.907	0.003
≤ 1,000 RMB	807 (15.62)	233 (28.87)		
> 1,000 RMB	4361 (84.38)	1044 (23.94)		
**Total**	5168 (100.00)	1277 (24.71)		

^†^ CCYL: Chinese Communist Youth League Communist Youth League; CPC: Communist Party of China

### Measurement model construction

The measurement model incorporated all hypothetical latent variables, namely sociodemographic characteristics, health status, knowledge about blood donation, and attitude toward blood donation. Following the elimination of observational variables with insignificant paths or low factor loadings, 14 variables were retained within the model, as outlined in Supplementary Table 1. Table 2 presents the *Spearman* correlation matrix of the retained variables, revealing significant correlations among many of them.

**Fig. 1:**
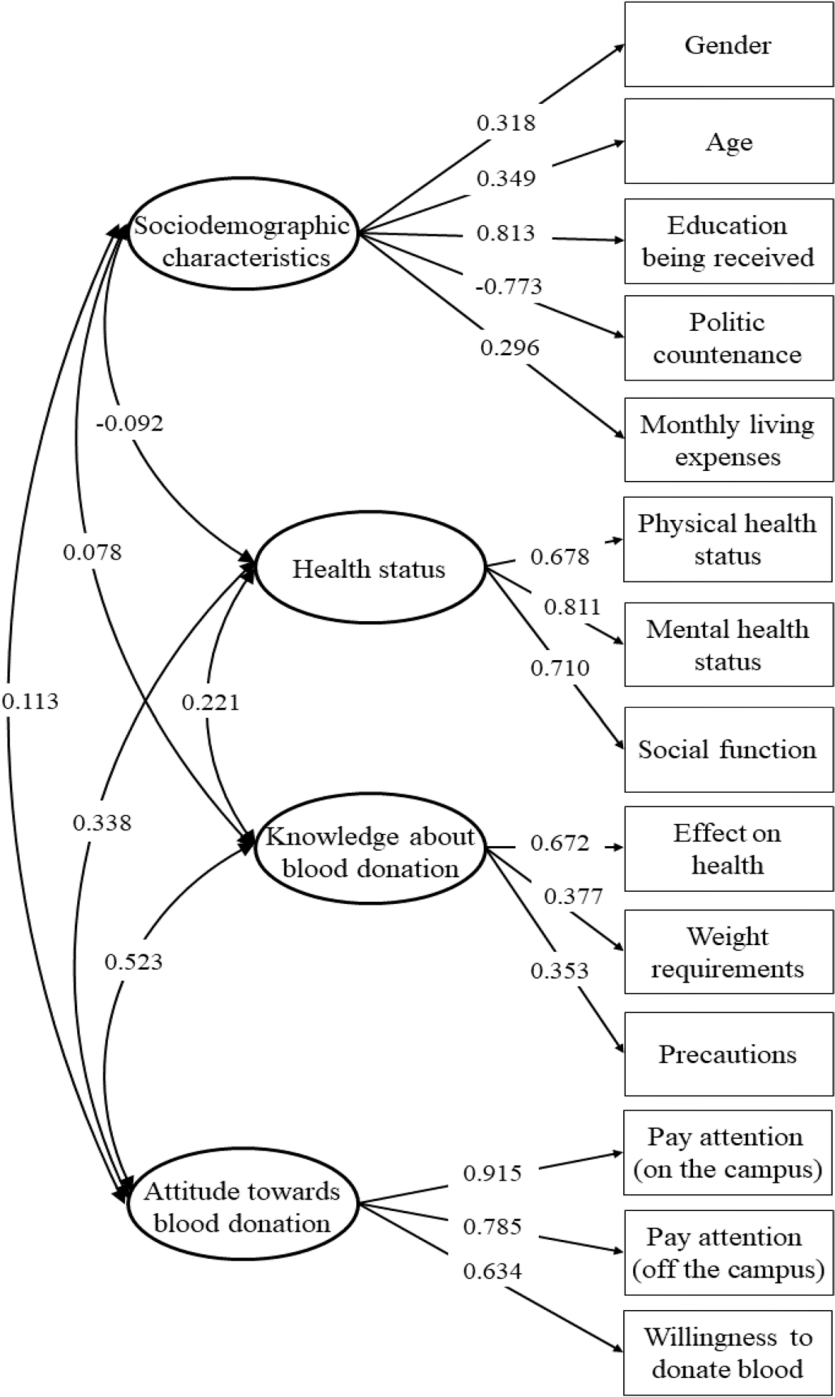
The measurement model of latent variables. Four latent variables and 14 manifest variables are connected by unidirectional paths with path coefficients on the lines. Four latent variables are connected by bidirectional arrow curves to each other with correlation coefficients on the lines. The variances are set to 1.000 during the model estimation and all the coefficients are significant

The final measurement model, depicted in Fig. 1, assigned five observational variables to sociodemographic characteristics, with factor loadings ranging from 0.296 to 0.813. Health status, knowledge about blood donation, and attitude towards blood donation were assigned three observational variables, with factor loadings ranging from 0.678 to 0.811, 0.353–0.672, and 0.634–0.915, respectively—all of which were statistically significant (*P* < 0.001). More so, the four latent variables exhibited significant intercorrelations (*P* < 0.001), with knowledge about blood donation and attitude towards blood donation demonstrating the strongest association (*r* = 0.523), while sociodemographic characteristics displayed the least correlation with knowledge about blood donation (*r* = 0.078). More importantly, the measurement model demonstrated a good fit to the data, as indicated by the following goodness-of-fit indices: *χ*^2^ = 1703.513, *df* = 54, *P* < 0.001, CFI = 0.938, TLI = 0.920, and RMSEA = 0.067 (95% confidence intervals [CI] = 0.064–0.069).

**Table 2:** *Spearman* correlation matrix for study variables (*N* = 5168)

Variables^†^	1	2	3	4	5	6	7	8	9	10	11	12	13	14
1	1.000													
2	0.041^**^	1.000												
3	0.139^***^	0.195^***^	1.000											
4	-0.180^***^	-0.188^***^	-0.381^***^	1.000										
5	0.070^***^	-0.020	0.197^***^	-0.085^***^	1.000									
6	-0.104^***^	-0.048^***^	-0.032^*^	0.020	0.018	1.000								
7	-0.061^***^	-0.038^**^	-0.033^*^	0.043^**^	0.030^*^	0.498^***^	1.000							
8	-0.007	-0.020	-0.016	0.022	0.054^***^	0.364^***^	0.505^***^	1.000						
9	-0.046^***^	0.017	0.070^***^	-0.044^**^	-0.012	0.077^***^	0.076^***^	0.110^***^	1.000					
10	-0.038^**^	-0.035^*^	-0.055^***^	-0.012	-0.020	0.066^***^	0.077^***^	0.008	0.043^**^	1.000				
11	0.101^***^	-0.018	0.019	-0.037^**^	0.025	0.031^*^	0.043^**^	0.001	0.036^**^	0.358^***^	1.000			
12	0.040^**^	0.066^***^	0.098^***^	-0.093^***^	0.023	0.129^***^	0.136^***^	0.232^***^	0.263^***^	0.086^***^	0.068^***^	1.000		
13	-0.034^*^	0.004	-0.024	-0.005	-0.026	0.106^***^	0.121^***^	0.242^***^	0.216^***^	0.061^***^	0.023	0.648^***^	1.000	
14	-0.035^*^	0.015	0.117^***^	-0.054^***^	-0.001	0.196^***^	0.156^***^	0.177^***^	0.291^***^	0.071^***^	0.073^***^	0.479^***^	0.383^***^	1.000

^†^ Variables: (1) Gender; (2) Age; (3) Education being received; (4) Politic countenance; (5) Monthly living expenses; (6) Physical health status; (7) Mental health status; (8) Social function; (9) Effect of blood donation on health; (10) Weight requirements for blood donation; 11. Precautions before blood donation; 12. Pay attention to blood donation publicity on the campus; 13. Pay attention to blood donation publicity off the campus; 14. Willingness to donate blood

^*^P < 0.05; ^**^P < 0.01; ^***^P < 0.001

### Structural equation model

**Fig. 2:**
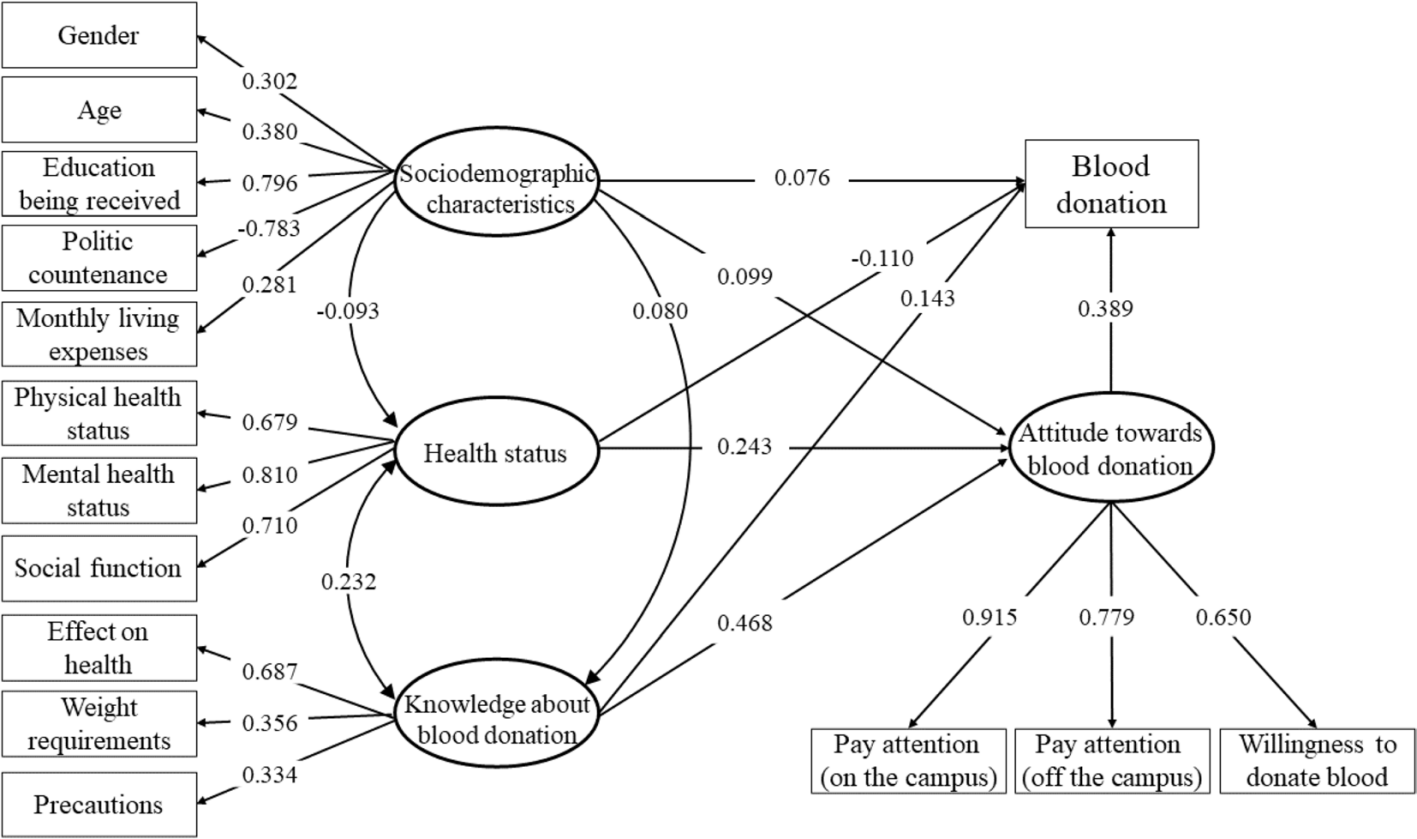
The structural equation model of sociodemographic characteristics, health status, knowledge about blood donation, attitude towards blood donation, and blood donation. The relationships of four latent variables, their corresponding manifest variables, and the associations of four latent variables with blood donation are presented. The standardized coefficients are shown on the paths, and except for one path that is dotted, all the coefficients are significant. The variances are set to 1.000 during the model estimation

Figure 2 illustrates the final SEM derived from the measurement model. Sociodemographic characteristics, health status, knowledge about blood donation, and attitude towards blood donation exhibited significant direct paths to blood donation, with regression coefficients of 0.076, -0.110, 0.143, and 0.389, respectively (*P* < 0.01). And sociodemographic characteristics, health status, and knowledge about blood donation demonstrated significant direct paths to attitude towards blood donation, with sociodemographic characteristics also exhibiting significant direct paths to health status and knowledge about blood donation.

Equally, the SEM displayed a good fit to the data, with *χ*^2^ = 2086.618, *df* = 81, *P* < 0.001, CFI = 0.927, TLI = 0.905, and RMSEA = 0.069 (95% confidence intervals [CI] = 0.067–0.072). In addition, Table 3 outlines the direct, indirect, and total associations of the four latent variables with blood donation. Notably, attitude towards blood donation demonstrated the strongest direct and total associations with blood donation. The variables identified in the estimated SEM collectively explained 22.22% of the variance in blood donation.

**Table 3:** Standardized associations of latent variables with blood donation

Variables	Association		
	Direct	Indirect	Total
Sociodemographic characteristics	0.076	0.059	0.135
Health status	-0.110	0.166	0.056
Knowledge about blood donation	0.143	0.178	0.321
Attitude towards blood donation	0.389	-	0.389

## Discussions

Blood serves as an indispensable medical resource, with blood donation forming the cornerstone of clinical transfusions. Remarkably, college and university students represent a crucial demographic of blood donors. This study pioneers an analysis of the prevailing blood donation landscape and potential influencing factors among college and university students in the Wuhan area. Our findings reveal direct associations between blood donation and sociodemographic characteristics, health status, blood donation cognition, and blood donation attitude, while sociodemographic characteristics, health status, and knowledge about blood donation demonstrate indirect associations. Notably, attitude towards blood donation emerges as the primary predictor of actual blood donation behavior.

### Blood donation rate

Wuhan boasts a college and university student population group exceeding one million, making it China’s foremost hub of tertiary education. Investigating blood donation trends among the students in this locale holds significant representative value. Our results indicate a blood donation rate of 24.71% among Wuhan’s college and university students, lower than that observed in larger urban centers like Beijing, 30.98% and Kunming, 41.63%, as well as neighboring regions such as Xinyang, 25.38% [27–29]. These disparities may stem from variations in blood donation promotion and recruitment strategies across regions, differing perceptions and attitudes toward blood donation among the students, or variances in research methodologies and participant demographics. Our findings underscore the untapped potential to invigorate college and university students’ enthusiasm for blood donation within this region.

### Sociodemographic characteristics

This section encompasses five variables: gender, age, education level, political outlook, and monthly living expenses. Our findings indicate a higher blood donation rate among female students compared to male students, aligning with some extant research [10, 15]. This trend may be attributed to females’ typically stronger empathy than males, motivating them toward blood donation [10]. Conversely, other findings suggest a lower blood donation rate among older females compared to males, potentially due to the challenges females face in donation eligibility stemming from childbirth and family responsibilities [30–32]. Furthermore, our study’s outcomes diverge from those in Qatar and Saudi Arabia, where religious constraints impede female blood donation [3, 33].

Notably, university students exhibit a higher blood donation rate compared to college students, consistent with existing research highlighting the correlation between higher education levels and increased likelihood of blood donation [32]. In a similar vein, elevated education levels tend to heighten awareness of blood donation, thereby promoting donation behavior [34].

Interestingly, our study reveals a positive correlation between the students’ blood donation rate and their monthly living expenses. This finding resonates with Lin’s research, indicating a linear relationship between socioeconomic status and blood donation rate [5]. Studies indicate that individuals with higher socioeconomic status are more inclined towards altruistic behaviors, providing theoretical support for our findings [35].

### Health status

Students in good self-assessed physical condition exhibit a higher blood donation rate compared to those in poor condition. Numerous studies cite failure to meet blood donation requirements and fear of the process as primary deterrents among college and university students [10, 33]. In our study, students with poor self-assessed health may perceive their physical condition as unsuitable for blood donation or harbor concerns about adverse reactions, thus refraining from donating blood. From a mental health standpoint, altruistic students display a heightened willingness to shoulder social responsibility, coupled with lower anxiety and fear towards blood donation, potentially fostering greater donation propensity [36, 37]. In the early stages of the study, we anticipated a positive correlation between better health conditions and higher blood donation rates; however, this was not confirmed by our results. Notably, the direct and indirect effects of health status on blood donation appear inconsistent, suggesting a potential masking effect between them. This finding implies a non-linear correlation between health status and blood donation, warranting further exploration. We speculate that students in good health may overly prioritize their health, leading to heightened apprehension toward blood donation and hindering their willingness to donate. This underscores the necessity of health education initiatives targeting blood donation among college and university students.

### Knowledge about blood donation

Numerous research findings, including our own, underscore the significant impact of blood donation knowledge levels on college students’ blood donation behaviors [38]. A substantial portion of students refraining from blood donation cite fears and concerns regarding safety, privacy, and potential health impacts [36, 39, 40]. Consequently, blood banks need to implement widespread, comprehensive dissemination of blood donation knowledge, particularly emphasizing its safety and health benefits, in a manner tailored to resonate with young people, thereby alleviating negative emotions stemming from misconceptions [41].

### Attitude toward blood donation

Our research highlights attitude towards blood donation as the primary determinant of blood donation among college and university students, in line with previous studies [42, 43]. Christopher et al.’s research demonstrates that a positive attitude towards blood donation not only facilitates blood donation but also effectively mitigates adverse reactions [44]. Studies grounded in the Theory of Planned Behavior (TPB), a prominent psychological framework in blood donation research, affirm that TPB-based interventions focused on promoting altruism, reducing anxiety, and fostering supportive social environments can bolster blood donation rates [43, 45].

### Strengths and limitations

Given the declining willingness among college and university students to donate blood, researching the influencing factors of blood donation in this pivotal demographic becomes imperative for formulating future recruitment strategies tailored to this population group. Through a comprehensive large-scale survey, this research investigates numerous potential influencing factors, including health status, elucidating the structural relationships that exist between multiple variables and blood donation. However, several limitations warrant consideration. Firstly, this survey adopts a cross-sectional design, precluding the determination of exact causal relationships. Additionally, discrepancies between predicted and observed results were noted during the analysis, highlighting the need for further validation of the relationship between health status and blood donation. Future studies could employ longitudinal designs to explore causal relationships and delve deeper into the nuanced influences on blood donation behavior among college and university students, potentially uncovering additional factors that may impact donation rates.

## Conclusions

This study utilizes SEM to delineate the structural relationships of sociodemographic characteristics, health status, knowledge about blood donation, and attitude toward blood donation on college and university students’ blood donation behaviors, underscoring the pivotal role of attitude toward blood donation as the primary predictor of donation behavior, and the mediating role of health status. These insights provide valuable guidance for the development of targeted recruitment strategies aimed at improving knowledge about blood donation, and fostering a culture and positive attitudes of blood donation among college and university students, ultimately contributing to the enhancement of blood donation rates and addressing the ongoing challenges in blood supply.

### Electronic supplementary material

Below is the link to the electronic supplementary material.

Supplementary Material 1

Supplementary Material 2

## Data Availability

The datasets used during the current study are available from the corresponding author on reasonable request.

## Abbreviations

CFI: Comparative fit index
CFA: Confirmatory factor analysis
CI: Confidence intervals
RMSEA: Root mean square error of approximation
SEM: Structural equation modeling
SRHMS-V1.0: Self-rated Health Measurement Scale—the Revised Version 1.0
TLI: Tucker–Lewis’s index
WLSMV: Weighted least squares with mean and variance
