# Supplementary material for: Factors associated with blood donation among college and university students in Wuhan, China: structural equation model
Source: BMC Public Health. 2024 Jul 10;24:1847. doi: 10.1186/s12889-024-19384-y (PMC11238382; doi:10.1186/s12889-024-19384-y)
Supplement: Supplementary file 1 — Supplementary Material 1 [file 12889_2024_19384_MOESM1_ESM.docx]

**Questionnaire about knowledge, attitude and practice of blood donation and influencing factors among college and university students in Wuhan, China**

Dear students

Hello!

Blood donation is a noble public welfare, and college and university students are high-quality blood donors. Wuhan blood center is conducting a survey about the knowledge, attitude and practice of blood donation and influencing factors among college and university students in Wuhan, with the purpose of improving blood donation in colleges and universities. Please spare 5-10 minutes to complete the questionnaire.

This survey was conducted anonymously. All information is strictly confidential and only used for scientific research. Thank you for your support!

**Part I Sociodemographic characteristics**

1. Your gender is:

A. Male

B. Female

2. Your nationality is:

A. Han

B. Minority nationality

3. Your age is: ________ years old.

4. The name of your college/university is: _________

5. Your educational background is:

A. Junior college student

B. Undergraduate student

C. Master’s student

D. PhD student

6.Your major is:

A. Liberal arts

B. Science

C. Engineering

D. Agronomy

E. Medicine

7.Your politic countenance is:

A. CPC member

B. Member of the Communist Youth League of China

C. Member of other democratic parties

D. Masses

8．Have you ever joined a school association or served as a class cadre?

A. Joined the school-level Association.

B. Joined the academy-level Association.

C. Served as a class cadre.

D. None of the above.

9. Your type of Hukou is:

A. Rural

B. Urban

10. Are you from a one-child family?

A. Yes

B. No

11. How much is your monthly living expenses?

A. ≤1,000RMB

B. 1,001-2,000 RMB

C. 2,001-3,000 RMB

D. ＞3,000 RMB

12. The social media apps you commonly use to browse information is:

A. Douyin

B. Weibo

C. Wechat

D. Bilibili

E. Little Red Book

F. Douban

G. Others

H. None of the above.

13. Your father's educational level is:

A. Primary school and below

B. Junior high school

C. High school or specialized secondary school

D. Junior College

E. Undergraduate college or above

14. Your father's politic countenance is:

A. CPC member

B. Member of other parties

C. Masses

15. Your Mather's educational level is:

A. Primary school and below

B. Junior high school

C. High school or specialized secondary school

D. Junior College

E. Undergraduate college or above

16. Your Mather's politic countenance is:

A. CPC member

B. Member of other parties

C. Masses

17. Are your parents medical workers?

A. My father is.

B. My mother is.

C. Both

D. Neither

18. Have your parents ever donated blood?

A. My father has.

B. My mother has.

C. Both

D. Neither

19. Your family's annual income is:

A. Less than 100,000 RMB

B. 100,000 RMB and above, less than 300,000 RMB

C. 300,000 RMB and above, less than 500,000 RMB

D. 500,000 RMB and above

**Part II Health Status**

Self-rated Health Measurement Scale (SRHMS)

(Jing X, Meng H, Li Y, Lu L, Yao Y. Associations of Psychological Capital, Coping Style and Emotional Intelligence with Self-Rated Health Status of College Students in China During COVID-19 Pandemic. Psychol Res Behav Manag. 2022 Sep 14;15:2587-2597. Doi: 10.2147/PRBM.S383743IF: 4.3 Q1. PMID: 36133628; PMCID: PMC9482953.)

**Part III Knowledge about blood donation**

68. How much do you know about blood donation?

A. Very familiar.

B. Familiar.

C. A little familiar.

D. Basically unfamiliar.

E. Completely unfamiliar.

69. Do you think blood donation has an effect on health?

A. Yes, it’s good for health.

B. Yes, it’s harmful to health.

C. No.

D. Uncertain.

70. Which is the age range for blood donation in China?

A. 15-55 years old

B. 18-55 years old

C. 15-60 years old

D. 18-65 years old

71. How long is the interval of whole blood donation in China?

A. 1 month

B. 3 months

C. 6 months

D. 12 months

72. How long is the interval of platelet donation in China?

A. 7 days

B. 14 days

C. 28 days

D. 3 months

73. How much do you know about the reimbursement policy for blood donation in Wuhan or other regions?

A. Very familiar.

B. Familiar.

C. A little familiar.

D. Basically unfamiliar.

E. Completely unfamiliar.

74. When is World Blood Donor Day?

A. May 14th

B. June 14th

C. July 14th

D. August 14th

75. What is the range of blood volume for each donation?

A. 100-200ml

B. 200-400ml

C. 300-400ml

D. 400-600ml

76_1(Male reviewer). Which is the weight requirement for male blood donors?

A. 45kg and above

B. 50kg and above

C. 55kg and above

D. 60kg and above

76_2(Female reviewer). Which is the weight requirement for female blood donors?

A. 40kg and above

B. 45kg and above

C. 50kg and above

D. 55kg and above

D. 55kg and above

77. As for the precautions before blood donation, which of the following is wrong?

A. The meal before blood donation should be light, and don’t donate blood on an empty stomach.

B. Be healthy during the week before blood donation, without cold, fever, diarrhea, taking drugs, etc.

C. Female should avoid menstruation and 5 days before and after menstruation.

D. Get enough sleep the day before blood donation, and sleep time should not be less than 6 hours.

78. As for the precautions after blood donation, which of the following is right?

A. Take more rest within 24 hours to avoid fatigue and strenuous exercise.

B. Eat more than usual, due to blood loss.

C. Don’t drink more water, in case of fainting.

D. Protect the cleanliness of the puncture needle hole. Have tub bath to avoid fatigue.

79. How do you get information about blood donation? (1-3 options are available, in order.)

A. Social media apps.

B. Street public service advertisements.

C. Street blood donation points.

D. Publicity of blood donation in school.

E. Family, friends, and classmates.

F. Books.

G. Hospital.

H. Traditional media such as TV and radio.

**Part IV Attitude towards blood donation**

80. Have you paid attention to the blood donation points on the campus before?

A. Yes, always.

B. Yes, usually.

C. Yes, sometimes.

D. Yes, but rarely.

E. No, never.

81. Have you paid attention to the blood donation points off the campus before?

A. Yes, always.

B. Yes, usually.

C. Yes, sometimes.

D. Yes, but rarely.

E. No, never.

82. Have you ever thought about donating blood before?

A. Yes, always.

B. Yes, usually.

C. Yes, sometimes.

D. Yes, but rarely.

E. No, never.

83.Are you willing to donate blood in the future?

A. Yes, very much.

B. Yes, I am.

C. It depends. (Skip to question 87)

D. No, not really. (Skip to question 87)

E. No, not at all. (Skip to question 87)

84. The main reason why you are willing to participate in blood donation is (1-3 options can be selected, in order):

A. Motivated by family, friends and classmates.

B. Do a meaningful thing on a special day.

C. Give love and save lives.

D. The psychological satisfaction and sense of honor brought by blood donation.

E. Blood donation souvenirs.

F. Get credits.

G. Enjoy the blood reimbursement policy.

H. You or people around you have transfused blood, so you want to repay the society by donating blood.

I. It’s good for health.

85. What kind of blood do you prefer to donate?

A. Whole blood

B. Platelet

C. Both.

86. How often do you prefer to donate blood?

A. According to the blood donation interval.

B. Every six months.

C. Every year.

D. Between one and two years.

E. Two years and above.

(Skip to question 88)

87. The main reason why you don't have strong willingness to donate blood is (1-3 options can be selected, in order):

A. Skeptical about the safety of blood donation.

B. Inconvenience, such as place and appointment process.

C. Dissatisfied with blood donation policies and benefits.

D. Personal physical reasons.

E. No time.

F. Family disagree.

G. I don't think it's necessary.

88. What is your parents' attitude towards blood donation?

A. Strongly supportive

B. Supportive

C. neutral

D. Unsupportive

E. Strongly unsupportive

89. Will you encourage relatives and friends to donate blood if their physical condition permits?

A. Yes, very much.

B. Yes, I will.

C. It depends. (Skip to question 91)

D. No, not really. (Skip to question 91)

E. No, not at all. (Skip to question 91)

90. The main reason why you are willing to encourage relatives and friends to donate blood is (1-3 options can be selected, in order):

A. It’s good for Health.

B. Give love and save lives.

C. Blood donation souvenirs.

D. Enjoy the blood reimbursement policy.

E. You or people around you have transfused blood, so you want to repay the society by donating blood.

(Skip to question 92)

91. The main reason why you will not encourage relatives and friends to donate blood is (1-3 options can be selected, in order):

A. Skeptical about the safety of blood donation.

B. Inconvenience, such as place and appointment process.

C. Dissatisfied with blood donation policies and benefits.

D. Dissatisfied with the experience of blood donation.

E. They have no time.

F. Relatives and friends themselves don’t support blood donation.

G. I don't think it's necessary.

92. Which kind of publicity and recruitment method of blood donation is better in your opinion?  (1-3 options can be selected, in order):

A. Propaganda on the campus, such as distributing leaflets and posting posters.

B. Social media.

C. Live broadcast and short videos.

D. Social practices, such as visiting blood stations.

E. Others.

**Part V Blood donation Behavior**

93. Have you ever donated blood?

A. Yes. (Skip to question 95)

B. No.

94. The main reason why you haven’t donated blood is (1-3 options can be selected, in order):

A. Skeptical about the safety of blood donation.

B. Inconvenience, such as place and appointment process.

C. Dissatisfied with blood donation policies and benefits.

D. Personal physical reasons.

E. No time.

F. Family disagree.

G. I don't think it's necessary.

H. Others.

(Skip to question 112)

95. How many times have you donated blood? ______________

96. The age of your first blood donation is _______ years old.

97. What kind of blood did you donate for the first time?

A. Whole blood

B. Platelet

98. The main reason why you decided to donate blood for the first time was (1-3 options can be selected, in order):

A. Motivated by blood donation points on the campus.

B. Motivated by street blood donation points.

C. Motivated by street public service advertisements.

D. Motivated by the blood donation promotion on the social media apps.

E. Motivated by family, friends and classmates.

F. Do a meaningful thing on a special day

G. Give love and save lives.

H. The psychological satisfaction and sense of honor brought by blood donation.

I. Blood donation souvenirs.

J. Get credits.

K. Enjoy the blood reimbursement policy.

L. You or people around you have transfused blood, so you want to repay the society by donating blood.

M. It’s good for health.

99. When was your last blood donation?

A. Within half a year.

B. Between half a year and one year.

C. 1 year and above, less than 2 years.

D. 2 years and above.

100. In most cases, which is your blood donation form?

A. Take the initiative to donate blood at blood donation points off the campus.

B. Participate in blood donation points on the campus.

C. Accidentally encounter street blood donation points.

D. Accidentally encounter blood donation points in school.

101. What is your blood donation habit?

A. Donate according to legal interval.

B. Donate according to own fixed interval, such as half a year.

C. Donate on some meaningful days (such as birthday, graduation, etc.)

D. Donate in organized blood donation activities.

E. Donate casually.

102. Where do you usually donate blood?

A. Street blood donation points.

B. School blood donation points.

C. Donate platelet at Wuhan blood center or Jiangnan sub center.

103. What kind of blood do you donate the most?

A. Whole blood

B. Platelet

104. Refer to the following criteria for the classification of blood donors, choose which kind you belong to:

A. Regular blood donor: have donated blood for ≥ 3 times, and at least once in the last year. (Skip to question 105)

B. Lapsed blood donor: have donated blood before, but not donated within the past 2 years. (Skip to question 106)

C. Return blood donor: donated blood again after becoming lapsed blood donors, and the donation time was within the past 2 years. (Skip to question 107)

D. Repeat blood donor: have donated blood for ≥ 2 times, and do not meet the requirements of regular, lapsed, return and first-time blood donors. (Skip to question 108)

E. First-time blood donor: donated blood for the first time, and the time of donation in the past 2 years. (Skip to question 109)

105. The main reason why you adhere to blood donation is (1-3 options can be selected, in order):

A. Motivated by family, friends and classmates.

B. Give love and save lives.

C. Satisfied with the experience of blood donation.

D. The psychological satisfaction and sense of honor brought by blood donation.

E. Blood donation souvenirs.

F. Get credits

G. Enjoy the blood reimbursement policy.

H. You or people around you have transfused blood, so you want to repay the society by donating blood.

I. It’s good for health.

J. Others.

(Skip to question 109)

106. The main reason why you didn't adhere to blood donation was (1-3 options can be selected, in order):

A. Skeptical about the safety of blood donation.

B. Inconvenience, such as place and appointment process.

C. Dissatisfied with blood donation policies and benefits.

D. Dissatisfied with the experience of blood donation.

E. Personal physical reasons.

F. No time

G. family disagree

H. I don't think it's necessary.

I. Others.

(Skip to question 109)

107. The main reason why you didn’t donate blood for at least 2 years was (1-3 options can be selected, in order):

A. Skeptical about the safety of blood donation.

B. Inconvenience, such as place and appointment process.

C. Dissatisfied with blood donation policies and benefits.

D. Dissatisfied with the experience of blood donation.

E. Personal physical reasons.

F. No time

G. family disagree

H. I don't think it's necessary.

I. Others.

108. The main reason why you donate blood again is (1-3 options can be selected, in order):

A. Motivated by blood donation points on the campus.

B. Motivated by street blood donation points.

C. Motivated by street public service advertisements.

D. Motivated by the blood donation promotion on the social media apps.

E. Motivated by family, friends and classmates.

F. Do a meaningful thing on a special day

G. Give love and save lives.

H. Satisfied with the experience of blood donation.

I. The psychological satisfaction and sense of honor brought by blood donation.

J. Blood donation souvenirs.

K. Get credits.

L. Enjoy the blood reimbursement policy.

M. You or people around you have transfused blood, so you want to repay the society by donating blood.

N. It’s good for health.

109. In general, are you satisfied with the blood donation services (both on and off the campus) in Wuhan?

A. Yes, strongly satisfied.

B. Yes, satisfied.

C. Neutral. (Skip to question 111)

D. No, dissatisfied. (Skip to question 111)

E. No, strongly dissatisfied. (Skip to question 111)

F. Haven’t donated blood in Wuhan. (Skip to question 111)

110. What is the main reason for your satisfaction? (1-3 options can be selected, in order):

A. Simple and straightforward blood donation process.

B. Excellent professional skills.

C. Provision of food for blood donors.

D. Various blood donation souvenirs.

E. Short time for blood donation.

F. Comfortable place for blood donation.

G. Good service attitude.

H. Others.

(Skip to question 112)

111. What are the main reasons for your dissatisfaction? (1-3 options can be selected, in order):

A. Complicated blood donation process.

B. Poor professional skills.

C. Unpalatable food supplied to blood donors.

D. Bad blood donation souvenirs.

E. Long time for blood donation.

F. Limited blood donation space.

G. Poor service attitude.

H. Others.

112. What aspects of blood donation do you think that need to be improved? (1-3 options can be selected, in order):

A. Increase the publicity of blood donation.

B. Simplify the process of blood donation.

C. Improve professional skills of staffs.

D. Improve the service of blood stations.

E. Enrich blood donation souvenirs.

F. Increase preferential policies for blood donors (such as taking bus, subway, etc.)

G. Improve the social status of blood donors.

H. Others.
