# Supplementary material for: Factors associated with blood donation among college and university students in Wuhan, China: structural equation model
Source: BMC Public Health. 2024 Jul 10;24:1847. doi: 10.1186/s12889-024-19384-y (PMC11238382; doi:10.1186/s12889-024-19384-y)
Supplement: Supplementary file 2 — Supplementary Material 2 [file 12889_2024_19384_MOESM2_ESM.docx]

**Supplementary Table 1 Assignment of study variables**

| **Variables** | **Value** |
| --- | --- |
| **Sociodemographic characteristics** |  |
| 1. Gender | 1 = Male, 2 = Female. |
| 2. Age | 1 = 18 years old, 2 = 19 years old, 3 = 20 years old, 4 = ≥21 years old. |
| 3. Education being received | 1 = Junior college, 2 = Undergraduate college. |
| 4. Politic countenance | 1 = Members of the CCYL or CPC**^†^**, 2 = Other parties or masses. |
| 5. Monthly living expenses | 1 = ≤1000RMB, 2 = >1000RMB. |
| **Health status** |  |
| 6. Physical health status | 1 = Very poor, 2 = Poor, 3 = Sub-health,4 =Good, 5 = Very good. |
| 7. Mental health status | 1 = Very poor, 2 = Poor, 3 = Sub-health,4 =Good, 5 = Very good. |
| 8. Social function | 1 = Very poor, 2 = Poor, 3 = Sub-health,4 =Good, 5 = Very good. |
| **Knowledge about blood donation** |  |
| 9. Effect of blood donation on health | 0 = Wrong, 1 = Right. |
| 10. Weight requirements for blood donation | 0 = Wrong, 1 = Right. |
| 11. Precautions before blood donation | 0 = Wrong, 1 = Right. |
| **Attitude towards blood donation** |  |
| 12. Pay attention to blood donation publicity on the campus | 1 = Never, 2 = Seldom, 3 = Sometimes, 4 = Frequently, 5 = Always. |
| 13. Pay attention to blood donation publicity off the campus | 1 = Never, 2 = Seldom, 3 = Sometimes, 4 = Frequently, 5 = Always. |
| 14. Willingness to donate blood in the future | 1 = Not at all, 2 = Not really, 3 = It depends, 4 = Willing, 5 = Very willing. |
| **Blood donation behavior** | 0 = No, 1 = Yes. |

^†^ CCYL: Chinese Communist Youth League Communist Youth League; CPC: Communist Party of China
